# Evaluation of Trace Metal Profile in *Cymbopogon validus* and *Hyparrhenia hirta* Used as Traditional Herbs from Environmentally Diverse Region of Komga, South Africa

**DOI:** 10.1155/2016/9293165

**Published:** 2016-10-04

**Authors:** Babalwa Tembeni, Opeoluwa O. Oyedeji, Ikechukwu P. Ejidike, Adebola O. Oyedeji

**Affiliations:** ^1^Department of Chemistry, Faculty of Science and Agriculture, University of Fort Hare, P.O. Box X1314, Alice 5700, South Africa; ^2^Department of Chemistry, School of Applied and Environmental Sciences, Walter Sisulu University, Mthatha 5099, South Africa

## Abstract

FAAS was used for the analysis of trace metals in fresh and dry plant parts of* Cymbopogon validus *and* Hyparrhenia hirta* species with the aim of determining the trace metals concentrations in selected traditional plants consumed in Eastern Cape, South Africa. The trace metal concentration (mg/kg) in the samples of dry* Cymbopogon validus *leaves (DCVL) showed Cu of 12.40 ± 1.000; Zn of 2.42 ± 0.401; Fe of 2.50 ± 0.410; Mn of 1.31 ± 0.210; Pb of 3.36 ± 0.401 mg/kg, while the samples of fresh* Hyparrhenia hirta *flowers (FHHF) gave Cu of 9.77 ± 0.610; Zn of 0.70 ± 0.200; Fe of 2.11 ± 0.200; Mn of 1.15 ± 0.080; Pb of 3.15 ± 0.100 mg/kg. Abundance of metal concentrations follows the order: Cu > Fe > Pb > Mn > Zn in the flower samples of* Cymbopogon validus* and* Hyparrhenia hirta* species. The concentrations of trace metals in both plant parts were below the permissible limits (PL) set by WHO. It is suggested that pharmacovigilance be carried out periodically to improve the quality, safety, and efficiency of various herbal products.

## 1. Introduction

Medicinal plants have played an indisputable role in the development of human race and culture, for example, religions and different ceremonies. Majority of cultures (Chinese, African, and Indian) around the world use plants directly as medicines for disease treating purposes. Medicinal plants are plants that contain substances that could be used by man because of their ability to exert a modifying benefit to the physiology of sick mammals [1] or plants that have component parts which are used as natural therapies. Virtually 80% of the populace in emerging nations depends heavily on the use of these plants/or products for primary healthcare purpose [2]. Therefore, medicinal plants are key resources in the creation of new drugs. Medicinal plants (MPs) are directly used as pharmaceuticals and/or as building blocks or starting materials for the production of semisynthetic drugs and, hence, have been playing a pivotal role in the design and development of potent therapeutic agents [3].

Medicinal plants can be used as blueprints for the production of synthetic drugs of a similar structure, for instance, the plant alkaloid Cocaine extracted from* Erythroxylum coca*, which has provided the chemical structure for the synthesis of Procaine and other related anaesthetics [4]. MP's therapeutic agent discovery continues to provide novel and imperative pointers against copious pharmacological targets comprising cancer, malaria, cardiovascular diseases, and neurological disorders which are reliable source of antimicrobial compounds, thus being used as antibiotics, and because of their chemical constituents such as triterpenoids, sesquiterpenes, flavonoids, fatty acids, and alkaloids [5]. Complementary and alternative medicine has received international consideration in the last few years with an occurrence of 31.4% populace in industrialized societies, 42–69% in United States, 71% in Canada, and 90% in UK, who are consuming nutritional supplements or natural health foodstuffs (minerals, vitamins, essential fatty acids, herbal products, amino acids, traditional Chinese medicines, homeopathic medicines, and probiotics) for treatment, including eradication of ailment triggering agents, avoidance of side effects, and improving the overall quality of life [6, 7].

The species* Cymbopogon validus* belongs to the family Poaceae.* Cymbopogon* species occur in large quantities in tropics and subtropics with unlimited scattering ranging from mountains and grasslands to dry lands [8]. Its species have countless uses in pharmaceuticals, cosmetics, food and flavour, and agricultural industries. The essential oils of* Cymbopogon* species are in large demand worldwide due to their wide industrial uses which include raw materials for confectionery, perfumery, and pharmaceuticals [9] and these essential oils and decoction are often used as a vermifuge, emetic, antirodent, anti-infective, and antispasmodic or to treat morning sickness [8, 9]. In Eastern and Southern Africa, it is used as a thatching material [10]. The genus* Hyparrhenia* belongs to the family Poaceae; it is a genus of 55 grass species such as (*Hyparrhenia anamesa*,* Hyparrhenia arrhenobasis*,* Hyparrhenia barteri*,* Hyparrhenia coleotricha*, and* Hyparrhenia confinis*).

Many of these grass species are commonly acknowledged as thatching grasses [10]. They are mostly native to tropical Africa and the Mediterranean. These are annual and perennial bunch grasses. The grass grows in a variation of habitat types, including dry conditions, heavy, eroded soils, and rocky and disturbed areas [11]. The initial flavonolignans glycosides to be isolated as natural products from* Hyparrhenia hirta* using high performance liquid chromatography (HPLC) were the rare diastereoisomeric flavonolignans tricin 4′-O-(erythro-beta-guaiacylglyceryl)ether and tricin 4′-O-(threo-beta-guaiacylglyceryl)ether together with their 7-O-glucosides [11].* In vitro* studies of flavonolignans have been established to have a wide variety of biological and pharmacological actions comprising antiallergic, antimicrobial (antifungal, antibacterial, and antiviral), anti-inflammatory, antioxidant, anticancer, and antidiarrhoea activities, alongside the capability to induce DNA mutation in the mixed lineage leukaemia gene [8, 9, 11].

Medicinal plants that are grown on polluted fields, near roadways or metal mining sites, possess greater chances of amassing heavy metals from the soil, water, and air. In general, the topography, the geochemical soil features, impurities in the soil, water, and air, and other development, transport, and storage conditions can significantly affect the properties and the quality of herbal plants and their formulations [12]. High levels of heavy metal contamination in the environment is increasing because of human activities such as agricultural practices, industrial processes, and natural causes such as weathering of rocks. Eventually, these heavy metals enter the plants through the soils and aerial fallouts and accumulate in these medicinal plants [13, 14]. The heavy metal contamination of herbal therapies is a present issue within traditional, complementary, and alternative medicines in numerous Asian, South American, and African herbal products [15, 16]. According to Khan et al. (2008), heavy metal concentrations differ in different plant parts and are influenced by whether a plant is grown in the wild or in cultivated environment [17].

In view of this, the study seeks to evaluate the levels of trace metals (iron, zinc, copper, manganese, and lead) present in different parts of the medicinal plants (*Cymbopogon validus* and* Hyparrhenia hirta*) collected from environmentally diverse region of Komga road near King William's Town, Eastern Cape of South Africa, using flame atomic absorption spectrometry (FAAS). The medicinal plants are used for variety of biological and pharmacological activities amongst the populace.

## 2. Materials and Methods

### 2.1. Reagents and Chemicals

Analytical grade reagents, chemicals, and deionised water were utilized all through this research.

### 2.2. Plant Materials

Fresh and dry* Cymbopogon validus* and fresh and dry* Hyparrhenia hirta* (totally 8 samples) were collected from Komga road (roadside) near King William's Town. Taxonomic identification of* Cymbopogon validus* and* Hyparrhenia hirta* was done by Dr. T. Dold and the specimens voucher was deposited in Selmar Schonland Herbarium Grahamstown (GRA) at Rhodes University. The voucher number was PR/Pl 03. The plant sample parts are leaves and flowers for both fresh and dry plants. The plants were then air-dried, ground to be in a powder form, and stored in fastened air tight bottles for further investigation. Trace metal analysis was carried out according to AOAC [18] using flame atomic absorption spectroscopy in four samples of the medicinal plants using wet digestion method [19].

### 2.3. Preparation of Extracts

All glassware and digestion vessels were soaked overnight in 20% nitric acid and washed with deionised water. Standard solutions of lead (Pb), manganese (Mn), iron (Fe), zinc (Zn), and copper (Cu) were obtained by dilution of 1000 mg/L stock solutions (Merck, Germany) with 5% nitric acid (HNO_3_) solution. The standardization curve for each element was linear by plotting the measured absorbance and concentration (mg/kg) and a correlation coefficient was achieved. Accurately, 1.00 g of plant samples (pulverized) was weighed and placed into a digestion tube; 30 mL of 55% nitric acid (HNO_3_) and 10 mL of 32% hydrochloric acid (HCl) in 3 : 1 mole ratio were added [20]. This mixture was heated for 3-4 hours until solubilisation of the sample was complete [20].

After cooling, the mineral solution was filtered through Whatman filter paper and volume was made to the mark with deionised water into a 25 mL volumetric flask. All the samples were analysed in triplicate using Flame Atomic Absorption Spectrophotometer (Thermo Fisher Scientific) Model Ice 3500 with Shimadzu, Wizard software. Optimized operating conditions for FAAS (Flame Atomic Absorption Spectrophotometer) of various heavy metals are recorded in Table 1. In order to ascertain the precision and accuracy of the analytical instrument, all necessary requirements were put in place during the analysis. Also, samples were duplicated as replication enhances the quality of the results. Blank and standard solutions were used to calibrate the instruments.

### 2.4. Statistical Analysis

All experiments were completed in triplicate for precision and accuracy of the results. Concentration of each metal was obtained from the calibration functions of each replicate and articulated in mg/kg on a dry/fresh-weight basis of the plant sample and results were reported as mean values ± standard deviation (mean ± SD). ANOVA was used for statistical analysis in the study (*p* < 0.05).

## 3. Results and Discussion

**Figure 1:**
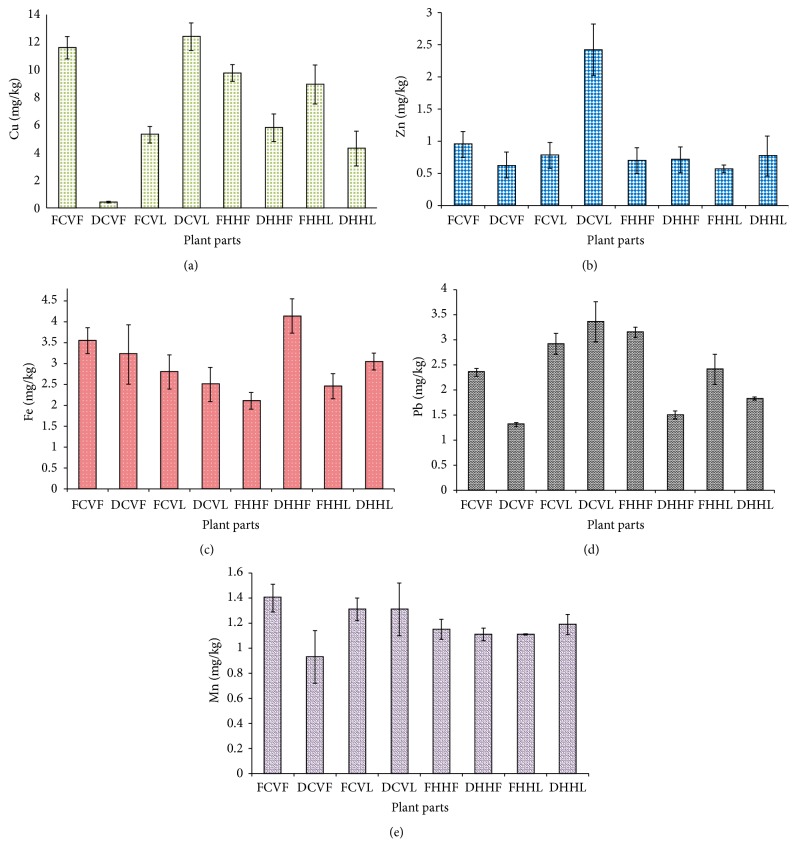
Concentration levels of trace metals ((a)–(e)) in selected plant parts species.

The atmosphere and soil have been continuously polluted with chemicals and heavy metals due to dynamic development of urbanization alongside widespread use of pesticides and fertilizers. In turn, these pollutants and heavy metals find their way to be deposited in the plant parts growing within the polluted areas and afterwards enter the human food chain via edible plant parts and/or extracts [21]. Results of analysis of five heavy metals (Pb, Mn, Fe, Zn, and Cu) analysed in leaf and flower of 8 samples of* Cymbopogon validus* and* Hyparrhenia hirta* medicinal plant species are summarized and displayed in Tables 2 and 3 and Figure 1, respectively.

The mean trace metal concentration (mg/kg) in the samples of* Cymbopogon validus *flowers (CVF) were found to be as follows: Cu: 6.01 ± 0.530; Zn: 0.79 ± 0.010; Fe: 3.39 ± 0.280; Mn: 1.17 ± 0.071; Pb: 1.83 ± 0.028 mg/kg;* Cymbopogon validus *leaves (CVL) showed the following: Cu: 8.85 ± 0.281; Zn: 1.60 ± 0.141; Fe: 2.65 ± 0.010; Mn: 1.31 ± 0.085; Pb: 3.14 ± 0.135 mg/kg, while in samples of* Hyparrhenia hirta *flower (HHF) the following was observed: Cu: 7.79 ± 0.275; Zn: 0.71 ± 0.020; Fe: 3.13 ± 0.148; Mn: 1.13 ± 0.021; Pb: 2.33 ± 0.014 mg/kg;* Hyparrhenia hirta *leaves (HHL) gave the following: Cu: 6.62 ± 0.106; Zn: 0.67 ± 0.177; Fe: 2.76 ± 0.071; Mn: 1.15 ± 0.053; Pb: 2.12 ± 0.192 mg/kg. Abundance of metal concentrations follows the following order: Cu > Fe > Pb > Mn > Zn in both flower samples of* Cymbopogon validus* and* Hyparrhenia hirta *while Cu > Pb > Fe was observed for leave samples of both medicinal plant species. The results indicate the presence of copper, lead, and iron in moderate concentrations; Zn and Mn recorded the lowest concentration in samples taken from Komga road.

Among the medicinal plant species, the average concentration of Cu was the highest in* Cymbopogon validus *leaves (8.85 ± 0.281 mg Cu/kg) followed by* Hyparrhenia hirta *flower (7.79 ± 0.275 mg Cu/kg) and the lowest concentration was found in* Hyparrhenia hirta *leaves for Zn (0.67 ± 0.177 mg Zn/kg) followed by* Hyparrhenia hirta *flower (0.71 ± 0.020 mg Zn/kg). The accumulation of metals by roots, leaves, and flowers increases with increasing available metal concentration in the biota [22]. Also, there is a relationship between the elemental profiles in the medicinal plant and their traditional remedial usage; this should be of paramount importance [23]. Contamination of traditional medicines by heavy metals is of major concern because of the toxicity, persistence, and bioaccumulative nature of such metals [24]. Even though WHO has formulated guidelines for quality assurance and control of herbal medicine, traditional practitioners lack enough knowledge which may result in medication with various types of heavy metal contamination.

High levels of copper concentrations were found in dry leaves of* Cymbopogon validus *12.40 ± 1.000 mg Cu/kg; fresh flowers of* Cymbopogon validus *contain 11.60 ± 0.810 mg Cu/kg, followed by fresh* Hyparrhenia hirta* flowers 9.77 ± 0.610 mg Cu/kg and fresh* Hyparrhenia hirta* leaves 8.94 ± 1.410 mg Cu/kg while very low concentration was observed in dry* Cymbopogon validus *flowers 0.42 ± 0.060 mg Cu/kg as displayed in Figure 1(a).

Copper is one of the crucial elements for plants and animal and essential mirconutrients for neutrological syetems and nervous system myelin sheaths [25]. Various metalloenzymes that are involved in haematopoiesis, vascular and skeletal tissues, catecholamine synthesis, and structure of nervous system are related to the significance of copper [25]; however, it can be toxic at excessive levels [26]. Copper concentration regulatory limits in herbal medicines are yet to be established by WHO/FAO [27]. However, China and Singapore had established limits for copper in medicinal plants at 20 and 150 mg/kg, respectively [27]. Comparing the metal perimeters in the studied selected plant part species with those proposed by China and Singapore, it was observed that all plant part species accumulated Cu below this limit.

Nevertheless, disproportionate ingestion of Cu could lead to the rupturing of erythrocytes and nephrotoxic effects; also, its constant consumption through food crops or herbal medicines may lead to the copper damage in human beings [13] leading to arthritis, growth impairment and reproductive performance, malnutrition, irregular hair growth and depigmentation, failure of the heart, and disturbances in gastrointestinal systems [21, 28].

Zinc is an important component of the human body but its excessiveness may be a suggestion of metal poisoning and growth impedance [29]. The highest level of zinc in both species is found in dry leaves of* Cymbopogon validus* (2.42 mg Zn/kg) and in low concentrations as it ranges from 0.63, 0.77, and 0.95 mg Zn/kg for dry* Cymbopogon validus *flowers, dry* Hyparrhenia hirta *leaves, and fresh* Cymbopogon validus *flowers, respectively, as seen in Tables 2 and 3 and Figure 1(b). The order of concentration of Zn can be as follows: DCVL > FCVF > DHHL = FCVL > DHHF > FHHF > DCVF > FHHL.

In general, the obtained results revealed that the plant part species studied had concentrations lower than 50 mg/kg, the FAO/WHO permissible limit stipulated for zinc in herbal medicines [12, 30, 31]. Zinc is in a state of competition for absorption with calcium nonheme iron and copper, due to the fact that the elements are all divalent and exist in living-Willian's series [32] and play a significant part in the synthesis, storage, and discharge of insulin [33].

The Fe concentration profile of medicinal plants part species used in the study are listed in Tables 2 and 3 and displayed in Figure 1(c). As observed from these tables, maximum level of Fe was found in dry flowers of* Hyparrhenia hirta *4.12 ± 0.410 mg Fe/kg and the lowest concentration of Fe was observed in fresh* Hyparrhenia hirta *flowers 2.11 ± 0.200 mg Fe/kg, including fresh flower of* Cymbopogon validus *3.53 ± 0.310 mg Fe/kg, dry flowers of* Cymbopogon validus *3.22 ± 0.710 mg Fe/kg, fresh leaves of* Cymbopogon validus *containing 2.80 ± 0.410 mg Fe/kg, and dry leaves of* Hyparrhenia hirta* containing 3.05 ± 0.201 mg Fe/kg (Figure 1(c)).

There is no permissible limit for iron in medicinal plants established yet by WHO [32, 33]; on the other hand, the permissible limit established for Fe by FAO/WHO [34] in edible plants was 20 mg/kg. When this proposed limit is compared with the iron concentrations in the medicinal plant part species under this study, it was observed to fall below the proposed permissible limit.

Fe is an indispensable element vital for plant photosynthesis, and it is essential for consistent blood supply in human body as iron (Fe) acts as a catalytic agent [35]. It also accelerates the oxidation of protein, carbohydrates, and fats to regulate body weight, an imperative dynamic in diabetes [17].

It is worth noting that excessive iron in the body can lead to prolonged effects that may be lethal for homeostasis [36]. Toxicity of Fe exhibits an antagonistic effect on different metabolic activities and cardiovascular system [37]. On the contrary, low iron content could bring about nose bleeding, gastrointestinal infection, and myocardial infarction [38]. The concentration of lead in the samples varied slightly in* Cymbopogon validus *flowers,* Cymbopogon validus *leaves,* Hyparrhenia hirta *flowers, and* Hyparrhenia hirta *leaves with average levels of 1.83 ± 0.028, 3.14 ± 0.135, 2.33 ± 0.014, and 2.12 ± 0.192 mg Pb/kg, respectively, as displayed in Figure 1(d).

The Pb levels can be found in the following order: DCVL > FHHF > FCVL > FHHL > FCVF > DHHL > DHHF > DCVF. The medicinal plant part species (flower and leaf) contained tremendously lower concentrations of Pb when compared to the permissible limit of 10 mg/kg established by WHO [31]. Lead is a nonessential element having no function in the human body or in plants, because it brings about acute and chronic poisoning and poses adverse effects on kidney, liver, and vascular and immune systems [39]. Human exposure to lead concentrations brings about reproductive dysfunction that exhibits biochemical-morphological features including decreased sperm quality, disorganized epithelia, and altered sperm morphology [21]. Constant usage of fertilizer, use of leaded gasoline, and sewage sludge are some of the major sources leading to intensification in Pb pollution in the ecosystem [7, 21].

Concentration of lead above permissible limits in medicinal plants and herbs has been reported in previous studies. Example of such is medicinal herbs in Jordan which were found to be 13.9, 13.1, and 16.9 mg Pb/kg on a dry weight basis, respectively [40]. Pb content of* Gunnera perpensa*, a medicinal plant from Mabandla Village, KwaZulu-Natal Province, South Africa, has been reported to have values above the permissible limit set by FAO/WHO [36] in edible plants [41]. Likewise in Egyptian and Iranian spices and medicinal plants, maximum lead levels of 14.4 and 21.7 mg/kg on a dry weight basis have been reported, respectively [42, 43].

Manganese is among the principal minerals associated with carbohydrate and fat metabolism [44]. The concentration of manganese in* Hyparrhenia hirta* and* Cymbopogon validus *is in an array of 0.93 ± 0.210 mg Mn/kg to 1.40 ± 0.110 mg Mn/kg for flowers of* Cymbopogon validus* and 1.11 ± 0.005 mg Mn/kg to 1.19 ± 0.080 mg Mn/kg for leaves of* Hyparrhenia hirta *as displayed in Tables 2 and 3 and Figure 1(e). The order of Mn concentration in the medicinal plant part species is as follows: FCVF > FCVL = DCVL > DHHL > FHHF > DHHF = FHHL > DCVF.

For medicinal plants, the WHO [31] limit is yet to be set for Mn; however, the permissible limit of Mn according to FAO/WHO [34] in edible plants is 2 mg/kg. In comparison of this limit with the results obtained from this study,* Cymbopogon validus *and* Hyparrhenia hirta *plant part species contain lower levels of Mn. Mtunzi and coworkers [41] reported* Berkheya setifera* collected from Mabandla Village, KwaZulu-Natal Province of South Africa, to accumulate Mn above the permissible limit on dry mass basis. A study by Sheded et al. [45] indicated that manganese levels in selected medicinal herbs of Egypt were in the range of 44.6 and 339 mg/kg while Jabeen et al. [46] reported a range of Mn variation with values between 32.64 mg/kg in* Justicia adhatoda* and 105.56 mg/kg in* Achyranthes aspera *on dry mass basis.

Mn is vital in regular reproductive activities and normal functioning of the central nervous system and also acts as cofactor for many enzymes. Elevated concentrations of manganese cause adverse effects mostly on the lungs and on the brain [47]. Its deficiency causes the disruption of blood supply to a part of the heart, triggering heart cells death [41]; it also causes myocardial infection, cardiovascular diseases, and disorder of bony cartilaginous growth in youngsters and leads to immunodeficiency disorder and rheumatic arthritis in adults [17, 38].

The results of FAAS analysis indicated that trace metals were present in varied concentrations in the eight samples of the traditional medicinal plant species commonly used in Eastern Cape, South Africa. The levels of trace metals in the plant species were of lower concentrations than internationally accepted permissible levels. Toxic metals accumulation varies significantly among plant species; toxic element uptake by plants is predominantly dependent on plant species and soil quality and its intrinsic controls [35]. Highly polluted locations are accountable for heavy metal accumulation in plant parts, consumed either raw or as finished herbal products [7].

## 4. Conclusion

Medicinal plant (MP) continues to play a crucial part, to a large extent, in the healthcare scheme of the world's populace. Fresh and dry* Cymbopogon validus* and* Hyparrhenia hirta* leaves and flowers contain trace metals: iron, copper, zinc, manganese, and lead in different degrees. The results indicate the presence of copper, lead, and iron in moderate concentrations; Zn and Mn recorded the lowest concentrations. The mineral contents in* Cymbopogon validus* parts for Cu and Zn are in the following order: DCVL > FCVF > FCVL > DCVF, whereas in* Hyparrhenia hirta* for Cu and Pb they are in the order: FHHF > FHHL > DHHF > DHHL. Comparison of the results with the defined permissible concentration limits by WHO showed that the levels of elements content in the locally available medicinal plant parts species under investigation fall below the permissible range and, hence, contribute no metal toxicity to the surrounding populace. Human health of the immediate users may not be affected by these medicinal plant part species when consumed orally or used for diet preparation. It is suggested that farming of herbal medicinal plants within the diameter closer to the vicinity of industries and high human activities be discouraged, as there might be probability of heavy metals uptake by medicinal plant parts, which will in turn end up in the food chain and finally to humans via herbs consumption. Furthermore, there should be a constant investigation into the effectiveness and safety measures of medicinal plants collected around anthropogenic activities while using the medicinal herbs for healthcare purposes.

## Figures and Tables

**Table 1:** Optimized operating conditions of FAAS for the analysis of trace metals in plant materials (leaves and flowers).

Elements	*λ*_max_ (nm)	Slit (nm)	Flame type	Flow rate (L/min)
Cu	324.8	0.5	Air/acetylene	1.1
Zn	213.9	0.2	Air/acetylene	1.2
Fe	248.3	0.2	Air/acetylene	0.9
Mn	279.6	0.5	Air/acetylene	1.1
Pb	283.3	0.5	Air/acetylene	1.2

**Table 2:** Trace metal concentration (mg/kg) in fresh and dry *Cymbopogon validus*.

Plant parts	Copper	Zinc	Iron	Manganese	Lead
FCVF	11.60 ± 0.810	0.95 ± 0.201	3.53 ± 0.310	1.40 ± 0.110	2.35 ± 0.080
DCVF	0.42 ± 0.060	0.63 ± 0.201	3.22 ± 0.710	0.93 ± 0.210	1.31 ± 0.040
FCVL	5.30 ± 0.602	0.78 ± 0.201	2.80 ± 0.410	1.31 ± 0.090	2.92 ± 0.210
DCVL	12.40 ± 1.000	2.42 ± 0.401	2.50 ± 0.410	1.31 ± 0.210	3.36 ± 0.401

FCVF: fresh *Cymbopogon validus* flowers; DCVF: dry *Cymbopogon validus* flowers; FCVL: fresh *Cymbopogon validus* leaves; DCVL: dry  *Cymbopogon validus* leaves.

**Table 3:** Trace metal concentration (mg/kg) in fresh and dry *Hyparrhenia hirta*.

Plant parts	Copper	Zinc	Iron	Manganese	Lead
FHHF	9.77 ± 0.610	0.70 ± 0.200	2.11 ± 0.200	1.15 ± 0.080	3.15 ± 0.100
DHHF	5.80 ± 1.000	0.71 ± 0.200	4.12 ± 0.410	1.11 ± 0.050	1.50 ± 0.080
FHHL	8.94 ± 1.410	0.57 ± 0.060	2.46 ± 0.301	1.11 ± 0.005	2.41 ± 0.301
DHHL	4.30 ± 1.260	0.77 ± 0.310	3.05 ± 0.201	1.19 ± 0.080	1.83 ± 0.030

FHHF: fresh *Hyparrhenia hirta* flowers; DHHF: dry *Hyparrhenia hirta* flowers; FHHL: fresh *Hyparrhenia hirta* leaves; DHHL: dry  *Hyparrhenia hirta* leaves.
